# The Enigmatic Kikuchi-Fujimoto Disease: A Case Report and Review

**DOI:** 10.1155/2014/648136

**Published:** 2014-02-06

**Authors:** Hassan Tariq, Vinaya Gaduputi, Arsalan Rafiq, Roopalekha Shenoy

**Affiliations:** Department of Medicine, Bronx Lebanon Hospital Center, 1650 Selwyn Avenue, Suit No. 10C, Bronx, NY 10457, USA

## Abstract

We report this case of a 33-year-old African American woman who presented to the clinic with preauricular and submandibular masses that she had noticed 6 weeks earlier. She gave a remote history of noticing bilateral cervical masses 3 years prior to this presentation that had not been investigated at the time and resolved spontaneously. Excisional biopsies of the cervical lymph nodes showed morphologic and immunophenotypic findings suggestive of Kikuchi Fujimoto disease (KFD). KFD is an uncommon, self-limited, and perhaps an underdiagnosed entity with an excellent prognosis. It mimics malignant lymphoma in presentation and therefore an accurate clinicopathological differentiation is crucial.

## 1. Introduction

Cervical lymphadenopathy could be a manifestation of a variegated group of illnesses ranging from benign infectious causes to malignant lymphomas. Kikuchi-Fujimoto disease (KFD), also called histiocytic necrotizing lymphadenitis, was initially described independently by Kikuchi and Fujimoto in 1972. It is a rare, benign, and self-limited syndrome of unknown etiology characterized by tender localized lymphadenopathy, constitutional symptoms such as fever and night sweats [1]. KFD patients are mostly young adults (mean age of diagnosis being 21) [2] with female preponderance (4 : 1) [3–5]. Diagnosis is confirmed histopathologically. It is generally a self-limited disease that regresses spontaneously without any specific therapy. It is an underdiagnosed condition with an excellent prognosis, making it imperative that it is differentiated from a malignant lymphoma. The awareness of this condition amongst clinicians and pathologists alike might help prevent misdiagnosis and inappropriate treatment [1]. This diagnosis should especially be considered in a young patient presenting with cervical lymphadenopathy with biopsy showing necrosis, fragmentation, and karyorrhexis [1, 3, 4, 6]. Recurrence is reported in about 4% of all cases of KFD. We present a case of KFD in a 33-year-old African American woman who had recurrent self-limited episodes of bilateral cervical lymph node enlargement.

## 2. Case Presentation 

A 33-year-old African American woman with no medical comorbidities and a recent trip to Jamaica presented to the clinic with complaints of multiple right-sided neck swellings. She reported developing laryngitis while she was in Jamaica, following which she noticed a right preauricular and a right submandibular mass that progressively increased in size. She also complained of fatigue, malaise, and intermittent chills. She had an episode of bilateral cervical lymphadenopathy 3 years earlier that spontaneously resolved. She had no prior history of tuberculosis or contact with it. She denied any history of smoking tobacco, drinking alcohol, or using illicit drugs. She denied taking any herbal or nonprescription medications at her house.

Physical examination revealed a comfortable and well-built young woman with anterior cervical, preauricular, submental, and right supraclavicular lymphadenopathy. The lymph nodes were firm and nontender. There was no hepatosplenomegaly, scleral icterus, or clinically appreciable lymphadenopathy elsewhere. Laboratory studies were significant for leucopenia with a total leucocyte count of 3200/*μ*L and an ANC (absolute neutrophil count) of 700/*μ*L. Patient was not anemic, with hemoglobin of 12.6 g/dL. Patient also had transaminitis with alanine aminotransferase of 116 U/L and aspartate aminotransferase of 64 U/L. Antinuclear antibody (ANA), rheumatoid factor (RF), and anti-DNA antibody were negative. Complement levels were within normal limits. Hepatitis-B surface antigen and neutrophil antibody assay were negative.

**Figure 1:**
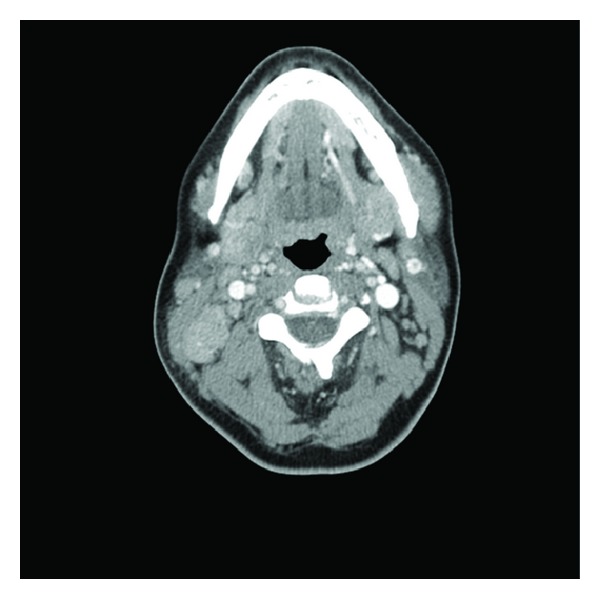
CT scan showing anterior and posterior cervical lymphadenopathy.

**Figure 2:**
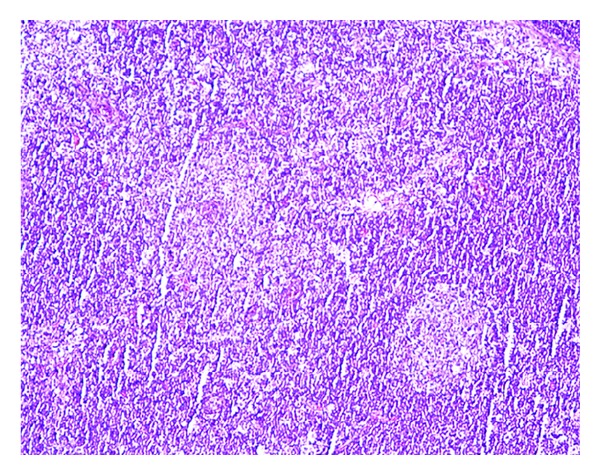
Lymph node showing reactive germinal centers and expanded paracortical areas suggestive of Kikuchi Fujimoto disease.

**Figure 3:**
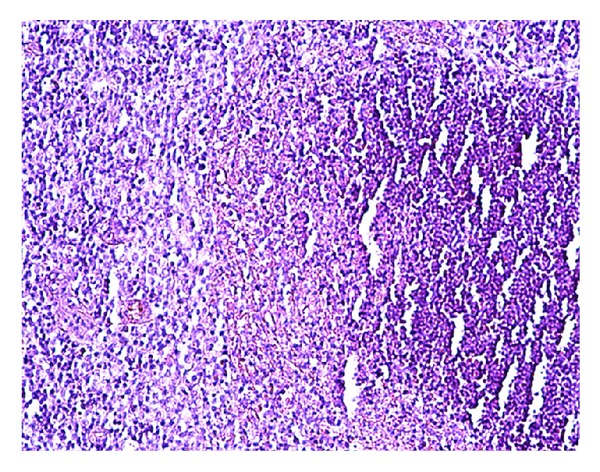
Multiple areas showing necrotic foci with apoptotic debris.

Chest X-ray was unremarkable. As the presentation was suspicious of lymphoma, a CT (computerized tomography) scan of neck, chest, abdomen, and pelvis was performed. It revealed asymmetric lymphadenopathy in the neck involving lymph nodes within the right posterior triangle (size of 2.8 cm), right internal jugular chain (size of 1.5 cm), bilateral submental lymph nodes (size of 1.2 cm), and right supraclavicular chain (size of 2 cm) (Figure 1). A fine needle aspiration biopsy of the lymph node showed mixed population of cells suggestive of a reactive process. An excisional biopsy of lymph node showed reactive follicles, an expanded parafollicular area (Figure 2) with clusters of large immunoblast-like cells, and histiocytes. Multiple areas showed necrotic foci with apoptotic debris (Figure 3). No granulocytes were present within these foci. Immunohistochemical staining showed CD3 positive T lymphocytes and CD20 positive B lymphocytes within the paracortical areas. Remnants of residual follicular dendritic cell meshworks and increased plasmacytoid dendritic cells were also found within the follicles. In situ hybridization for Ebstein-Barr virus encoded mRNA (EBER) was negative. These morphologic and immunophenotypic findings were suggestive of Kikuchi Fujimotos disease.

Patient was treated symptomatically with decrease in size of the lymph nodes gradually over the successive few weeks. Patient also experienced improvement in her fatigue and malaise. ANC improved to 1100/*μ*L. Her prior self-remitted episode of bilateral cervical lymphadenopathy 3 years prior was likely the first episode of KFD and the current presentation a possible relapse.

## 3. Discussion

KFD was initially reported in 1972 by Japanese pathologists Kikuchi and Fujimoto et al. independently. They described the disease as “lymphadenitis with focal proliferation of reticular cells accompanied by numerous histiocytes and extensive nuclear debris.” The incidence of KFD is unknown. It typically occurs in patients during third and fourth decade of life and is 3 to 4 times more common in women than in men. The disease is more prevalent in Asian populations. This geographic predominance may be related to the presence of certain HLA alleles such as HLA class II alleles, *HLA-DPA1* and *HLA-DPB1*, which are more prevalent in Asian KFD patients [7]. These genes are extremely rare or absent among Caucasians [1]. Although more prevalent in Asia, KFD has been observed in patients of all ages, genders, and races [8, 9]. It can involve both nodal and extranodal locations [3, 9].

There is much speculation about the etiology of KFD. An infectious or autoimmune cause has been suggested. Infectious agents such as *Yersinia enterocolitica*, *Brucella*, *Bartonella henselae*, *Entamoeba histolytica*, *Mycobacterium szulgai*, and *Toxoplasma gondii* were implicated but subsequent studies failed to support these findings [7, 10]. Viruses such as Epstein-Barr virus, herpes virus, cytomegalovirus, parvovirus, paramyxovirus, parainfluenza virus, Rubella, hepatitis-B, human immunodeficiency virus (HIV), human T-lymphotropic virus type-1 (HTLV-1), and the Dengue virus have all been suggested as probable etiologies of KFD, but never convincingly demonstrated [7]. On the other hand, some authors hypothesized that KFD may reflect a self-limited autoimmune condition induced by virus-infected transformed lymphocytes. This is because, histopathologically, findings of tubuloreticular structures in the lymphocytes and the endothelial cells in patients with SLE are similar to those found in patients with KFD. Clinically, KFD disease may mimic systemic lupus erythematosus (SLE) [1, 5, 10]. They share a common sex and age predisposition. They also have similar histological feature leading to speculation that KFD is a self-limited, SLE-like autoimmune condition caused by virus-infected transformed lymphocytes [10]. However, the association of KFD with SLE remains unclear [7].

The onset of KFD could be acute or subacute, evolving over two to three weeks. The main clinical feature of KFD is typically unilateral lymphadenopathy, with cervical involvement in 70% to 98% of cases [2, 4, 11]. The jugular lymph nodes and posterior cervical chain are most commonly involved [12]. However, any lymph node region can be involved including the axillary (14%) and supraclavicular (12%) nodal chains. The lymph nodes are usually small (less than 3 cm) and mobile. The lymphadenopathy may be firm and sometimes painful. Lymphadenopathy is usually isolated but few patients have generalized lymphadenopathy. Fever can be the first symptom in 30% to 50% of the cases [4]. Less frequent symptoms include weight loss, nausea, vomiting, sore throat, and night sweats. Skin lesions like maculopapular, morbilliform, urticarial rashes, or a disseminated erythema have been reported [3, 4].

A review of the literature suggests that neutropenia and an elevated erythrocyte sedimentation rate were the major abnormal hematological findings. A few patients had atypical lymphocytes in the peripheral smear [4] and rarely elevated aspartate aminotransferase or alanine aminotransferase levels were seen [13, 14]. Few patients diagnosed with KFD had positive laboratory test results for SLE and later developed clinically overt SLE. It is therefore recommended that KFD patients undergo long-term monitoring for the development of SLE, though data regarding frequency of follow-up visits and duration of the follow-up remains unclear. ANA, rheumatoid factor, and anti-ds DNA were negative in our patient.

This disorder does not have a characteristic radiological appearance [11]. The findings of CT and MRI (magnetic resonance imaging) in KFD can be variable and mimic not only lymphoma but also various nodal diseases with necrosis such as metastasis and tuberculosis. In a study of 96 retrospective CT scans of patients with confirmed KFD, Kwon et al. found that multiple homogeneous lymphadenopathies involving levels II to V were found in most, with 94% being smaller than 2.5 cm. This could allow some differentiation from lymphoma which typically produces few but larger nodes, perinodal infiltration, and necrosis [3, 12].

The definitive diagnosis of KFD is made through lymph node excision biopsy and histologic examination [11]. Kikuchi disease has been most commonly mistaken for malignant lymphoma. One study showed that 30% of the 108 lymph node biopsies reviewed were initially misdiagnosed as lymphomas [11, 15]. The histopathological features can be classified into three stages: (i) proliferative stage expressing various histiocytes, plasmacytoid monocytes, lymphoid cells containing karyorrhectic nuclear fragments, and eosinophilic apoptotic debris; (ii) necrotizing stage showing a degree of coagulative necrosis; and (iii) xanthomatous stage predominantly containing foamy histiocytes. The absence of granulocytes is also an important feature. The lack of monoclonal lymphocyte receptors rules out the possibility of a lymphoma [4]. Although histologic features are distinct in KFD, some overlaps exist, especially with SLE [1, 2, 9].

The differential diagnosis of a slow-growing neck mass is extensive, including malignant lymphoma, SLE, Hodgkin disease, toxoplasmosis, metastatic carcinoma, infectious mononucleosis, acquired immunodeficiency syndrome, cat-scratch disease, and angioimmunoblastic lymphadenopathy [4, 7, 9]. Considering the presentation of our patient, the diagnosis of a lymphoma was indeed high on our list of differentials and an excisional biopsy was performed to rule out this diagnosis. Although KFD is exceedingly uncommon in Western countries, it is necessary to consider it among differential diagnoses, as its treatment dramatically differs from that of lymphoma, tuberculosis, or SLE. The histological differential diagnoses of KFD include reactive lymphadenitis associated with SLE, herpes simplex and other microorganisms, non-Hodgkin lymphoma, plasmacytoid T-cell leukemia, Kawasaki disease, acute myeloid leukemia, and even metastatic adenocarcinoma [3, 9].

Kikuchi-Fujimoto disease is typically a self-limited disease that rarely requires specific treatment and resolves within one to four months [5, 11]. The course of cervical lymphadenopathy is benign and resolves spontaneously [5]. However, a recurrence such as that seen in our patient is reported in up to 3 to 4% of cases [2, 16]. No hereditary risk has been documented in KFD. Very few cases have been reported as fatal. The treatment is mainly supportive including analgesics (NSAIDs) and antipyretics to alleviate lymph node tenderness and fever. In case of extranodal disease with neurological involvement, the use of corticosteroids appears to improve the patient's condition rapidly. Use of hydroxychloroquine [17, 18], immunoglobulins [19], and minocycline [20] has been reported with excellent results in some cases [7].

Our case was unique as it represented a relapse of KFD in a non-Asian woman. Our patient had transaminitis and granulocytopenia with neutropenia that improved as the size of lymph nodes decreased. This gives an insight into the natural course of the disease. KFD is an uncommon, self-limited, and perhaps an underdiagnosed condition with an excellent prognosis. Unfortunately, the etiology, pathogenesis, diagnosis, and management of KFD still remain enigmatic and further research is required to answer these questions.
